# Comparison of nedaplatin and cisplatin in concurrent chemoradiotherapy for cervical cancer: a systematic review and meta-analysis

**DOI:** 10.1007/s10147-026-02968-6

**Published:** 2026-01-29

**Authors:** Maki Umemiya, Kazuhiro Kou, Yoshihide Inayama, Jun Kamei, Ken Yamaguchi, Yoshie Yamada, Takahiro Itaya, Yosuke Yamamoto, Masaki Mandai, Yusuke Ogawa

**Affiliations:** 1 Department of Gynecology and Obstetrics, Graduate School of Medicine, Kyoto University, Kyoto, Japan; 2 Department of Healthcare Epidemiology, Graduate School of Medicine, Kyoto University, Kyoto, Japan; 3 Department of Obstetrics and Gynecology, Graduate School of Biomedical and Health Sciences, Hiroshima University, Hiroshima, Japan

**Keywords:** Cervical cancer, Chemoradiotherapy, Cisplatin, Nedaplatin, Meta-analysis

## Abstract

**Background:**

Cisplatin-based concurrent chemoradiotherapy (CCRT) is the standard treatment for locally advanced cervical cancer; however, its nephrotoxicity and gastrointestinal toxicity often limit treatment eligibility and completion. Nedaplatin, a cisplatin analogue with reduced renal and gastrointestinal toxicity, has been increasingly used in East Asia, but its comparative efficacy and safety in cervical cancer have not been comprehensively evaluated.

**Methods:**

We systematically searched MEDLINE, Embase, CENTRAL, CNKI, Ichushi Web, ICTRP, and ClinicalTrials.gov for randomized controlled trials comparing nedaplatin versus cisplatin-based CCRT. The primary efficacy outcome was all-cause mortality at 3 years, and the primary safety outcome was renal toxicity. Secondary outcomes included mortality at 1 and 5 years, progression or mortality, hematologic and gastrointestinal toxicities, liver dysfunction, and quality of life. Random-effects meta-analyses were performed using risk ratios.

**Results:**

Seventeen trials met the eligibility criteria. All-cause mortality at 3 years did not differ significantly between the groups (RR 0.88; 95% CI 0.51–1.51; I^2^ = 0%). Nedaplatin significantly reduced renal toxicity (RR 0.25; 95% CI 0.20–0.31; I^2^ = 0%). Short-term outcomes favored nedaplatin, including lower 1 year mortality (RR 0.61; 95% CI 0.40–0.93) and fewer 1 year progression or mortality events (RR 0.63; 95% CI 0.44–0.91). The incidences of anemia and severe nausea/vomiting were also lower with nedaplatin. No eligible study assessed quality of life.

**Conclusion:**

Nedaplatin showed fewer adverse effects and comparable or improved short-term outcomes compared with cisplatin. These findings support nedaplatin as a potential alternative for patients who are cisplatin-intolerant or frail. Confirmation in large, high-quality trials with long-term follow-up and patient-reported outcomes is warranted.

**Supplementary Information:**

The online version contains supplementary material available at 10.1007/s10147-026-02968-6.

## Introduction

Cervical cancer is the 4th most common cancer among women worldwide. According to GLOBOCAN 2020, approximately 600,000 new cases and 340,000 deaths were reported globally [1]. Although the spread of HPV vaccination and cervical cancer screening has led to decline in cervical cancer incidence and mortality, rates remain high, especially in countries with low vaccine uptake; for example, in Japan, coverage has been relatively low due to delayed public promotion and safety concerns. [2].

For locally advanced cervical cancer, concurrent chemoradiotherapy (CCRT) is the standard treatment. CCRT has increasingly replaced surgery as the first-line treatment with advances in radiotherapy techniques [3]. Weekly cisplatin administration during radiotherapy is recommended by the NCCN guidelines [4] since it enhances radiosensitivity and improves survival. However, its nephrotoxicity, gastrointestinal toxicity including nausea/vomiting, and the need for intensive hydration limit its use in elderly patients, those with heart failure, or those with impaired renal function (e.g., malignant ureteral obstruction common in advanced cancer).

Nedaplatin, a cisplatin analog developed in Japan in 1983, was designed to reduce nephrotoxicity and gastrointestinal toxicity. The first report on its use in CCRT for cervical cancer was published in 2008 [5]. Its comparable efficacy and safety to cisplatin have been demonstrated in meta-analysis and large-scale randomized controlled trials (RCTs) for nasopharyngeal cancer [6–8].

A previous systematic review and meta-analysis in Chinese in 2020 focused mainly on comparing the safety profiles of nedaplatin and cisplatin in the treatment of cervical cancer [9]. However, no meta-analysis has assessed both efficacy and safety. Therefore, this study aims to assess the efficacy and safety of nedaplatin-based CCRT compared to those of cisplatin-based CCRT in patients with cervical cancer.

## Materials and methods

This systematic review and meta-analysis was conducted in accordance with the PRISMA guidelines [10]. A comprehensive search was performed in MEDLINE, Embase, CENTRAL, CNKI (China National Knowledge Infrastructure), Ichushi Web (Japan Medical Abstracts Society database), ICTRP, and ClinicalTrials.gov, using terms related to “cervical cancer”, “cisplatin”, “nedaplatin”, and “concurrent chemoradiotherapy”. The detailed search strategy is provided in Supplementary Table 1. The last search was conducted on March 27, 2025. No restrictions were placed on language or publication status.

We included randomized controlled trials (RCTs) that enrolled adult patients (aged 18 years or older) with cervical cancer. Eligible studies compared concurrent chemoradiotherapy using nedaplatin versus cisplatin. The primary efficacy outcome was all-cause mortality at 3 years and hazard ratios (HRs). The primary safety outcome was renal toxicity. Secondary outcomes included all-cause mortality at 1 and 5 years and progression or mortality at 1, 3, and 5 years, nausea/vomiting, liver dysfunction, hematologic toxicity, and quality of life (QOL). Adverse events were classified using the common terminology criteria for adverse events (CTCAE) version 5.0. Because severe hematologic and gastrointestinal toxicities are clinically most relevant, nausea/vomiting and hematologic events were extracted as Grade 3–4, whereas renal and hepatic toxicities—where even mild abnormalities may influence treatment decisions—were extracted as Grade 1–4. Since efficacy may differ between post-hysterectomy and non-hysterectomy settings, studies conducted in the post-hysterectomy setting were included only in the analysis of safety outcomes and excluded from the efficacy analysis. Two reviewers (MU and KK) independently screened the titles and abstracts of all identified records, followed by full-text review. Studies considered potentially eligible by either reviewer were retrieved for full-text assessment. Final inclusion decisions were also made independently by the 2 reviewers. Disagreements were resolved through discussion or consultation with a third reviewer (YO). Two reviewers (MU and KK) independently extracted data from the included studies. Risk of bias was assessed for the primary efficacy outcome (all-cause mortality at 3 years) using the Cochrane risk of bias tool [11]. Two reviewers (MU and JK) independently assessed the following domains: sequence generation, allocation concealment, blinding (participants, personnel, and outcome assessors), incomplete outcome data, selective outcome reporting, and other sources of bias. Discrepancies were resolved through discussion with a third reviewer (YO).

We contacted the corresponding authors of studies with missing outcome data to obtain additional information, but did not receive any responses.

We calculated risk ratios for binary outcomes. We also planned to conduct a meta-analysis using hazard ratios (HR) for time-to-event data if sufficient data were available. Heterogeneity was assessed using I^2^ statistics and examined visually using forest plots. All meta-analyses were conducted using a random-effects model with the inverse variance method. All statistical analyses and figure generation were performed using R software (version 4.3.3; R Foundation for Statistical Computing, Vienna, Austria).

## Results

**Fig. 1:**
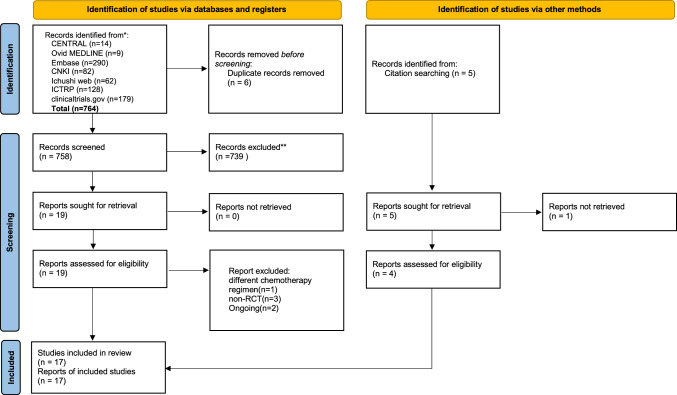
PRISMA 2020 flow diagram of the study

A total of 764 records were retrieved through database searches. After removing duplicates and screening titles and abstracts, 17 studies were included in the final analysis [12–28]. The PRISMA flow diagram is presented in Fig. 1.

One study [25] investigated CCRT following hysterectomy, and the remaining 16 studies focused on CCRT for locally advanced cervical cancer. The former was included only in the safety analysis, whereas the latter was included in both efficacy and safety outcome analyses. Renal and hepatic toxicities were assessed as Grade 1–4, whereas nausea/vomiting and hematologic toxicities were evaluated as Grade 3–4. The basic characteristics of the included studies are summarized in Table 1.

**Table 1:** List of selected randomized controlled trials

Author (publish year)		number of patients	Stage	Age	Histology	Planned chemotherapy cycle	Chemotherapy completion rate	Radiotherapy equipment/technique	Whole pelvic radiotherapy	Pelvic boost	Intracavitary brachytherapy	Notes	chemotherapy dose and regimen
Cheng 2011 [12]	nedaplatin	32	2B-3	< 70(median not reported	SCC^a^ 30/32	6	Not reported	15 MV^e^, Four-field	25–30 Gy (1.8–2.0 Gy/fraction, 5 ×/week)	15–20 Gy (1.8–2.0 Gy/fraction, 4 ×/week)	Ir-192 HDR^j^: 6 Gy × 6–7 (36–42 Gy)	No EBRT^k^ on BT days	30 mg/m2,weekly
	cisplatin	32		< 70(median not reported	SCC 29/32	6	Not reported						40 mg/m2,weekly
Lou 2011 [13]	nedaplatin	31	2B-4A	≦60 14	SCC 27/31	Not reported	≥ 4 in 25/31	Not specified	26 Gy + 24 Gy w/midline block (2 Gy, 3 ×/week)	Not reported	Ir-192 HDR: 4 Gy × 11 (44 Gy); vaginal BT 30–40 Gy	BT from week 3	40 mg/m2,weekly
	cisplatin	31		≦60 16	SCC 28/31	Not reported	≥ 4 in 17/31						40 mg/m2,weekly
Wang 2011 [14]	nedaplatin	34	2A-3B	median 56	SCC (all)	6	Not reported	6–15 MV	30–36 Gy (2.0 Gy/fraction, 5 ×/week)	16–20 Gy (2.0 Gy/fraction, 4 ×/week)	Ir-192 HDR: 6–7 Gy × 6–7 (36–46 Gy)	Field: L4 to 2 cm below obturator	40 mg/m2,weekly
	cisplatin	34		median 56.5	SCC (all)	6	Not reported						40 mg/m2,weekly
Bi 2013 [15]	nedaplatin	40	2B-4A	median 48	SCC 38/40	6	39/40	10 MV, LINAC^f^	36 Gy (1.8–2.0 Gy/fraction, 5 ×/week)	45–50 Gy (2.0 Gy/fraction, 4 ×/week, w/shield)	Ir-192 HDR: 6–7 Gy × 6–7 (36–46 Gy)	Weekly BT, no EBRT same day	30 mg/m2,weekly
	cisplatin	40		median 49	SCC 37/40	6	37/40						30 mg/m2,weekly
Duan 2013 [16]	nedaplatin	30	2B-3B	median 49.5	SCC 28/30	6	≥ 3 in 23/30	Box-field technique (energy not specified)	45–50 Gy (1.8–2.0 Gy/fraction, 25 fractions)	Midline block after 30 Gy	Ir-192 HDR: 6–7 Gy × 6 (Total point A dose: 36–42 Gy)	Not specified	30 mg/m2,weekly
	cisplatin	30		median 52.5	SCC 26/30	6	≥ 3 in 16/30						30 mg/m2,weekly
Ye 2013 [17]	nedaplatin	90	2B-3B	53 ± 9	SCC 83/90	6	Not reported	6 MV, 3D-CRT^g^	45 Gy/25 fractions (1.8 Gy/fraction)	Up to 61 Gy if nodal involvement	Ir-192 HDR: 6 Gy × 5	CT-based planning	30 mg/m2,weekly
	cisplatin	90		52 ± 10	SCC 85/90	6	Not reported						30 mg/m2,weekly
Cui 2014 [18]	nedaplatin	36	2A-3B	53.12 ± 4.59	SCC 31/36	6	Not reported	IMRT^h^ (6MV)	45 Gy (2.0 Gy/fraction, 5 ×/week), external RT 30 Gy	additional 15 Gy from BT plan	Ir-192 HDR: 5–7 Gy × 2/week × 5–6 weeks	CT-based planning	30 mg/m2,weekly
	cisplatin	36			SCC 29/36	6	Not reported						30 mg/m2,weekly
Li 2014 [19]	nedaplatin	48	2B-3B	48.5 ± 6.5	SCC 42/48	Not reported	Not reported	6–15 MV	40 Gy (2.0 Gy/fraction × 5/week × 4 weeks)	Four-field box with shield	During four-field phase	Details partially missing	40 mg/m2,weekly
	cisplatin	48		49.1 ± 7.6	SCC 44/48	Not reported	Not reported						40 mg/m2,weekly
Wang 2014 [20]	nedaplatin	35	3 ~ 4	58.3 ± 7.1	not reported	4	Not reported	Not specified	30 Gy (1.8–2.0 Gy/fraction, 5 ×/week)	20 Gy (4 ×/week)	6–7 Gy × 6 (40 Gy total)	weekly BT	40 mg/m2,weekly
	cisplatin	35		57.9 ± 7.6	not reported	4	Not reported						40 mg/m2,weekly
Zhao 2014 [21]	nedaplatin	24	2B-4A	55 ± 20	SCC (all)	5	24/24	6MV	40–45 Gy total (2.0 Gy/fraction, 5 ×/week)	Not specified	6–8 Gy × 6–7 (36–50 Gy total)	CT-based planning	25 mg/m2,weekly
	cisplatin	24		53 ± 15	SCC (all)	5	17/24						25 mg/m2,weekly
Shao 2015 [22]	nedaplatin	43	2B-3B	34.35 ± 8.77	SCC 39/43	6	Not reported	Not specified	≤ 30 Gy (1.8–2.0 Gy/fraction, 5 ×/week)	≤ 20 Gy (4 ×/week)	5 Gy × 2/week; total ≤ 50 Gy	BT from week 3	30 mg/m2,weekly
	cisplatin	43		35.06 ± 8.62	SCC 38/43	6	Not reported						30 mg/m2,weekly
Li 2016 [23]	nedaplatin	20	2A-3B	51.8 ± 6.2	SCC 38/40(possible reporting error)	6	Not reported	Liner accelerator (energy not specified)	50 Gy (180–200 cGy/fraction, 5 ×/week)	Not specified	Not specified	Not specified	30 mg/m2,weekly
	cisplatin	20		50.4 ± 7.3	SCC 37/40(possible reporting error)	6	Not reported						30 mg/m2,weekly
Liao 2017 [24]	nedaplatin	40	2B-3B	54.53 ± 8.49	SCC 38/40	6–7	≥ 4 in 37/40	8 MV	40 Gy (2.0 Gy/fraction, 5 ×/week)	Up to 56 Gy	5 Gy × 2/week × 5	Extended field L4/5–S2/3	40 mg/m2,weeklyx6-7
	cisplatin	40		54.45 ± 7.51	SCC 39/40	6–7	≥ 4 in 33/40						40 mg/m2,weeklyx6-7
Xiao 2019 [25]	nedaplatin	105	1B1-2B	48.5 ± 7.4	SCC (proportion not reported)	4–5	completed as planned	IMRT or 3D-CRT	45–50 Gy (1.8–2.0 Gy/fraction, 5 ×/week)	BT^i^ boost if margin-positive	6–7 Gy × 4–5	Postoperative	35–40 mg/m^2^, weekly × 5
	cisplatin	95		48.6 ± 6.4	SCC (proportion not reported)	4–5	completed as planned						35–40 mg/m^2^, weekly × 5
Wang 2019 [26]	nedaplatin	38	2B-3B	55 ± 8.3	SCC 36/38	4 and more	Not reported	8MV LINAC	40 Gy (2.0 Gy/fraction × 5/week)	16 Gy boost (2.0 Gy/fraction. 3x/week)	Co-60 HDR: 5 Gy × 2/week × 5	no EBRT on BT days	40 mg/m2,weekly
	cisplatin	38		54 ± 7.4	SCC 37/38	4 and more	Not reported						40 mg/m2,weekly
Yang 2022 [27]	Nedaplatin	78	1B-4A	median 52.9(30–82)	SCC 82.5%, Ad^b^ 13.7%, ASC^c^ 2.5%, SmCC^d^ 1.3%	0:2, 1:6, 2:13, 3:234:24, 5:12	≥ 4 in 36/80	6 MV	45–50.4 Gy (1.8 Gy/fraction × 25–28)	Not reported	Not described	Only EBRT	30 mg/m^2^, ≤ 5 cycles within ≤ 10-day intervals
	cisplatin	79		median 49.9(28–72)	SCC 82.5%, Ad 13.7%, ASC 2.5%, SmCC 1.3%	0:1, 1:9, 2:19, 3:30, 4:16, 5:5	≥ 4 in 21/80						40 mg/m^2^, ≤ 5 cycles within ≤ 10-day intervals
Xu 2023 [28]	nedaplatin	34	2B-3B	58.25 ± 3.06	not reported	5–6	Not reported	IMRT (15MV)	45–50 Gy (1.8–2.0 Gy/fraction, 5 ×/week)	20–30 Gy (with midline block)	6 Gy × 6	Not specified	30 mg/m2,weekly
	cisplatin	34		58.17 ± 3.21	not reported	5–6	Not reported						25 mg/m2,weekly

^a^*SCC* Squamous cell carcinoma, ^b^*Ad* Adenocarcinoma

^c^*ASC* Adenosquamous carcinoma, ^d^*SmCC* Small cell carcinoma

^e^*MV* Megavoltage, ^f^*LINAC* Linear accelerator

^g^*3D*-*CRT* 3-dimensional conformal radiotherapy

^h^*IMRT* Intensity-modulated radiotherapy

^i^*BT* Brachytherapy, ^j^*HDR* High-dose-rate

^k^*EBRT* External beam radiotherapy

### Risk of bias assessment

Risk of bias was assessed for the 5 studies that reported all-cause mortality at 3 year. Due to insufficient reporting, the risk of bias for blinding of participants and personnel was judged as “unclear” in 4 studies and “high” in one study, and allocation concealment was judged as “unclear” in 4. Consequently, several domains were classified as having an “unclear” risk of bias (Fig. S1).

### Primary efficacy outcome: all-cause mortality at 3 years and HR

**Fig. 2:**
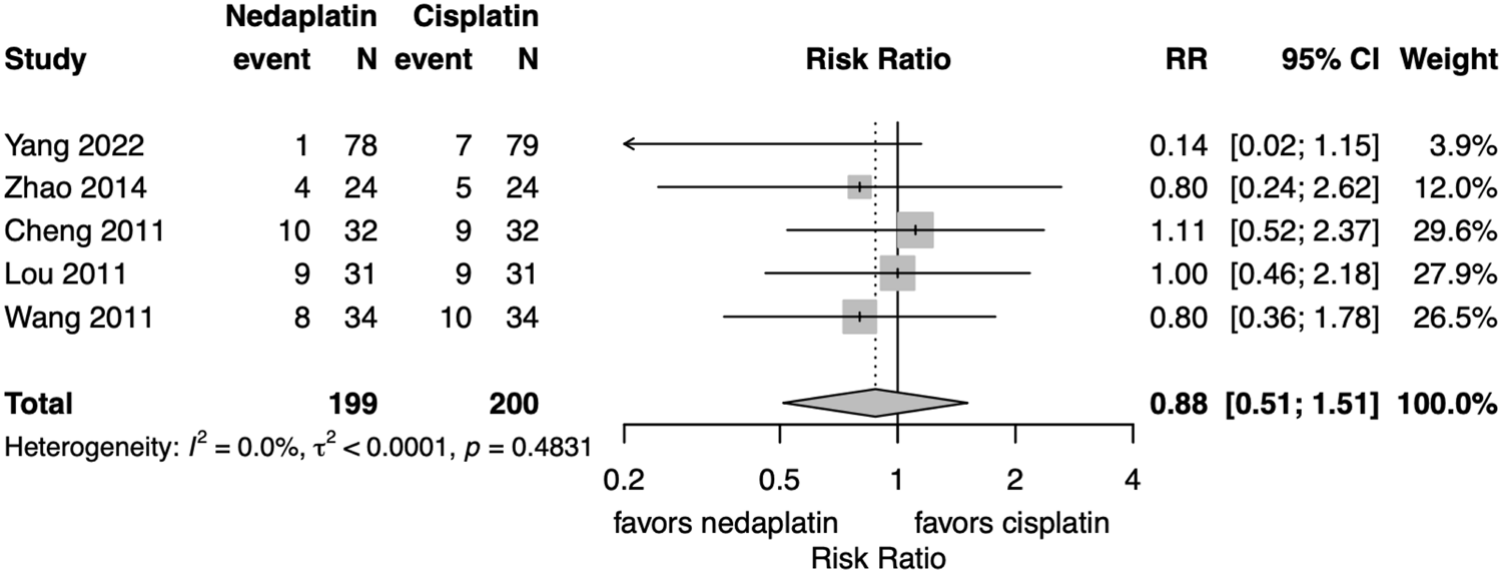
Forest plots for all-cause mortality at 3 years. *CI* confidence interval, *RR* risk ratio; Total represents the number of patients; Event represents mortality

Meta-analysis of 3 year all-cause mortality was not substantially different between the groups (5 studies, risk ratio 0.88, 95% CI 0.51–1.51, I^2^:0.0%) (Fig. 2). Meta-analysis using hazard ratio (HR) was not feasible due to the availability of only a single study, which reported an HR of 0.131 (95% CI 0.016–1.068), suggesting a potential survival benefit with nedaplatin, although the result did not reach statistical significance [27].

### Primary safety outcome: renal toxicity

**Fig. 3:**
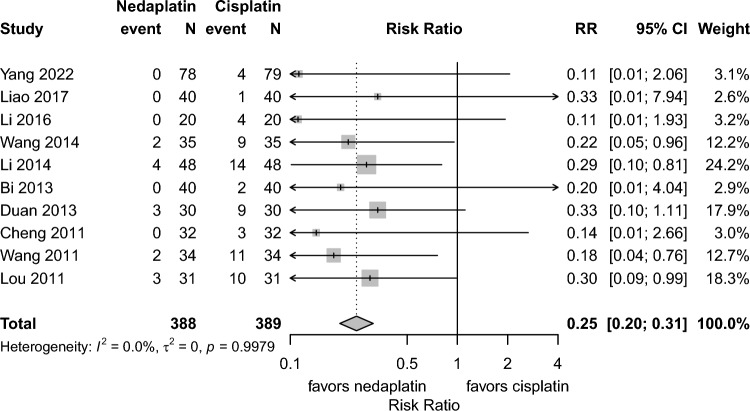
Forest plots for renal toxicity. *CI* confidence interval, *RR* risk ratio; Total represents the number of patients; Event represents renal dysfunction (Grade1-4)

Renal toxicity (grade 1–4) was fewer in the nedaplatin group than in the cisplatin group (10 studies, risk ratio 0.25, 95% CI: 0.20–0.31, I^2^:0.0%). (Fig. 3).

#### Secondary outcomes

**Fig. 4:**
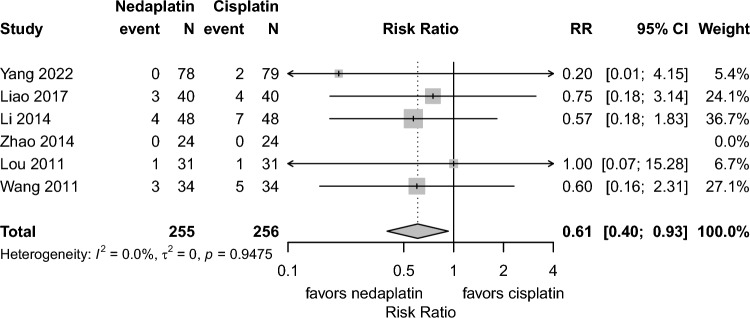
Forest plots for all-cause mortality at 1 year. *CI* confidence interval, *RR* risk ratio; Total represents the number of patients; Event represents mortality

**Fig. 5:**
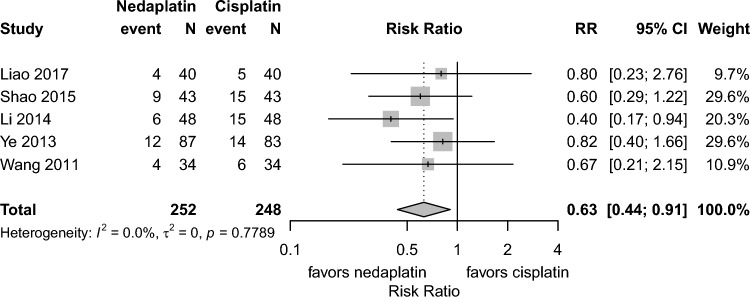
Forest plots for progression or mortality at 1 year. *CI* confidence interval, *RR* risk ratio; Total represents the number of patients; Event represents progression or mortality

- **All-cause mortality at 1 and 5 years**
  All-cause mortality at 1 year (6 studies, risk ratio 0.61, 95% CI 0.40–0.93, I^2^:0.0%) and progression or mortality at 1 year was lower (5 studies, risk ratio 0.63, 95% CI 0.44–0.91, I^2^:0.0%) in the nedaplatin group compared to the cisplatin group. (Fig. 4). No study reported all-cause mortality at 5 years.
- **Progression or mortality at 1, 3, and 5 years**
  The pooled analysis showed fewer 1 year progression or mortality in the nedaplatin group than in the cisplatin groups. (5 studies, risk ratio 0.63, 95% CI 0.44–0.91, I^2^:0.0%) (Fig. 5). As only one study [14] reported progression or mortality at 3 years, a meta-analysis was not feasible. In that study, the 3 year progression or mortality proportion was 35.3% in the nedaplatin group and 38.2% in the cisplatin group [14]. No study reported the 5 year progression or the mortality proportion
- **Nausea and vomiting:**
  Grade 3–4 nausea and vomiting occurred less frequently in the nedaplatin group than the cisplatin group (13 studies, risk ratio 0.38, 95% CI 0.29–0.50, I^2^:0.0%). (Fig. S2)
- **Liver dysfunction:**
  Despite relatively high heterogeneity among studies, no significant difference was observed between the 2 groups (5 studies, risk ratio 0.77, 95% CI 0.22–2.65, I.^2^:50.8%). (Fig. S3)
- **Hematologic toxicity:**
  Grade 3–4 anemia occurred less frequently in the nedaplatin group than the cisplatin group (6 studies, risk ratio 0.69, 95% CI 0.57–0.84, I^2^:0.0%) (Fig. S4). For leukopenia, no difference was observed (10 studies, risk ratio 0.88, 95% CI: 0.65–1.20, I^2^:0.0%) (Fig. S5). For thrombocytopenia, although heterogeneity was moderate (I^2^ = 55.3%), the risk ratio was 1.28 (95% CI 0.57–2.88) (Fig. S6).
- **Quality of life (QOL):**
  Although QOL was considered an important outcome, none of the included studies evaluated it.

## Discussion

The results of this study suggest that nedaplatin may be a reasonable alternative to cisplatin in concurrent chemoradiotherapy (CCRT) for cervical cancer. In terms of efficacy, although all-cause mortality at 3 years was not different between the groups, both 1 year progression and mortality of nedaplatin treatment were lower compared to the cisplatin group. In terms of safety, the incidences of renal toxicity, anemia, and nausea/vomiting were lower in the nedaplatin group, indicating a more favorable toxicity profile.

These findings may have clinical implications for treatment selection. In recent years, the use of CCRT has expanded, and with advances in radiation therapy techniques, CCRT is increasingly chosen over surgery as the first-line treatment [3]. This trend is supported by technological advancements in radiotherapy. Intensity-modulated radiation therapy (IMRT), as a foundational technique, has enabled more conformal dose delivery while reducing exposure to surrounding healthy tissue, contributing to improved survival outcomes [29]. Image-guided radiation therapy (IGRT) and MRI-based brachytherapy have further enhanced the precision and safety of treatment, leading to broader clinical indications for radiotherapy. Furthermore, the integration of artificial intelligence into radiotherapy planning holds promise for improving efficiency and standardization in treatment delivery, potentially accelerating and broadening the application of advanced radiotherapy techniques [30]

Nephrotoxicity associated with cisplatin is a major concern in administering CCRT. An increasing number of patients are being considered unsuitable for cisplatin due to age, comorbidities, or general frailty. Patients aged 65 years and older account for approximately 20–25% of all cervical cancer cases, and this proportion is increasing [31, 32]. More than 70% of elderly patients are diagnosed at an advanced stage [31], and due to delayed diagnosis and comorbidities, they often receive suboptimal treatment and have poorer prognoses compared to younger patients [33].

Nedaplatin may offer a safer and more feasible alternative to cisplatin for patients with heart failure requiring fluid restriction or postrenal failure due to advanced disease. Cisplatin -related toxicities, particularly nephrotoxicity, remain major barriers to treatment continuity in these populations. The use of nedaplatin may enable more patients to complete the planned treatment, potentially contributing to better treatment adherence and outcomes. Although carboplatin has also been explored as a substitute for cisplatin, some studies suggest that while it may reduce nephrotoxicity, it could result in inferior survival outcomes [34, 35]. In contrast, nedaplatin has the potential to reduce toxicity while maintaining or even improving survival outcomes, suggesting that it may be a more balanced alternative. Its clinical feasibility and tolerability in CCRT have also been reported previously, including in patients considered less suitable for cisplatin [36]. Its comparable efficacy and safety to cisplatin have been demonstrated in meta-analysis and large-scale randomized controlled trials (RCTs) for nasopharyngeal cancer [6–8].—and the present findings support its potential application in cervical cancer as a new therapeutic indication.

In our meta-analysis, short-term outcomes, including mortality and progression were superior in the nedaplatin group compared to the cisplatin group. One possible explanation of this result is the higher chemotherapy completion rate in the nedaplatin group. Among the studies included in our literature review, only 7 explicitly reported chemoradiotherapy completion rates and in all studies, the nedaplatin group demonstrated higher completion rates than the CDDP group, as shown in Table 1. Although a large RCT on nasopharyngeal carcinoma reported a higher completion rate in the CDDP group [7, 8], the distinct clinical characteristics of cervical cancer—including a higher frequency of postrenal dysfunction and the exclusively female patient population, who frequently experience nausea and vomiting—may make nedaplatin more tolerable and thus more likely to be completed than CDDP. Further research is warranted to comprehensively evaluate treatment completion in this context.

To our knowledge, this is the first meta-analysis to comprehensively compare the safety and efficacy of nedaplatin and cisplatin in CCRT for cervical cancer based on available RCTs. Previous reviews focused solely on safety and did not include some relevant studies identified in our search, suggesting a lack of comprehensive systematic literature search. Moreover, 3 additional RCTs have been published since then.

This study has several limitations. First, although the present meta-analysis suggests favorable short-term outcomes with nedaplatin-based CCRT, these findings should be interpreted with caution, as the number of available randomized trials remains limited and robust long-term survival data are lacking. Although the protocol aimed to evaluate 5 year survival, only short-term follow-up data (1–3 years) were available. Further long-term RCTs are needed. In addition, some outcomes (e.g., liver dysfunction and thrombocytopenia) showed moderate heterogeneity (I^2^ around 50%), reflecting possibly differences in the definitions of liver dysfunction (e.g., hepatic enzymes, biliary enzymes, bilirubin) or timing of assessments. Second, most included studies lacked clear reporting of blinding procedures, which may affect the reliability of the findings. Observer bias in the reporting of adverse events cannot be ruled out. Third, due to the limited number of included studies, we were unable to formally assess publication bias using funnel plots or statistical tests. Therefore, the possibility of selective publication or reporting bias cannot be excluded. Fourth, all included studies were conducted in China, reflecting the current use of nedaplatin mainly in East Asia. This geographic concentration may limit the generalizability of the findings to other populations and healthcare systems. Differences in patient characteristics, treatment protocols, supportive care, and regulatory environments across regions should be considered when interpreting these results and applying them to other clinical settings. This likely relates to its limited regulatory approval outside the region and the established role of nedaplatin worldwide. To assess its potential as an alternative to cisplatin, further trials outside East Asia are needed to evaluate its generalizability and to consider its adoption in other countries. Finally, a major limitation is the absence of QOL assessment. In recent years, clinical research has placed increasing importance not only on efficacy but also on QOL. The lack of QOL assessment is a major shortcoming. However, since nausea and vomiting, which are known to affect QOL [37], were significantly less common in the nedaplatin group, this may indirectly suggest better QOL in patients receiving nedaplatin.

Building on these limitations, future studies should aim to address remaining gaps. Large-scale RCTs with long-term follow-up of nedaplatin-based CCRT are warranted. Such trials should include subgroup analyses of elderly and renally impaired patients—populations traditionally considered difficult to treat—and should evaluate treatment completion rates, predictors of adverse events, and integrated analyses of multifaceted clinical outcomes, including QOL. Additionally, in low- and middle-income countries, where the burden of cervical cancer continues to rise, nedaplatin should be evaluated as a potentially simple and accessible regimen, and its cost-effectiveness should be considered an important area of future research.

## Supplementary Information

Below is the link to the electronic supplementary material.

Supplementary file1 (PDF 1973 KB)

Supplementary file2 (DOCX 18 KB)
