# Supplementary material for: Comparison of nedaplatin and cisplatin in concurrent chemoradiotherapy for cervical cancer: a systematic review and meta-analysis
Source: Int J Clin Oncol. 2026 Jan 29;31(3):537–47. doi: 10.1007/s10147-026-02968-6 (PMC12932270; doi:10.1007/s10147-026-02968-6)
Supplement: Supplementary file 1 — Supplementary file1 (PDF 1973 KB) [file 10147_2026_2968_MOESM1_ESM.pdf]

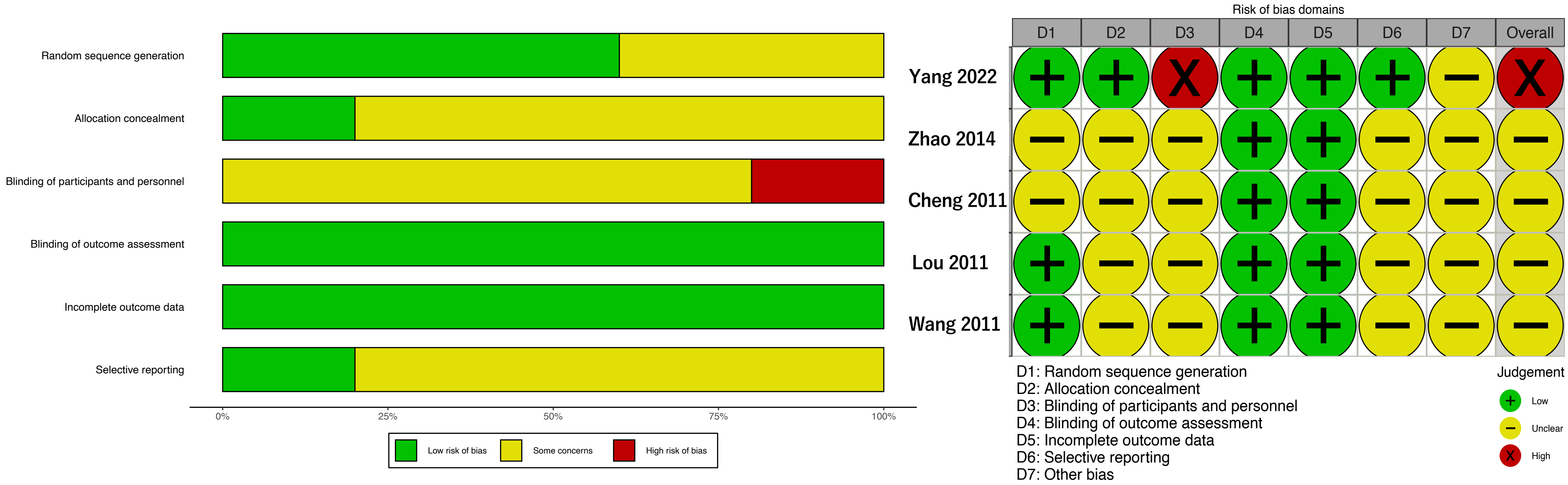
Random sequence generation
Allocation concealment
Blinding of participants and personnel
Blinding of outcome assessment
Incomplete outcome data
Selective reporting
0%
25%
50%
75%
100%
Low risk of bias
Some concerns
High risk of bias
Risk of bias domains
D1
D2
D3
D4
D5
D6
D7
Overall
Yang 2022
Zhao 2014
Cheng 2011
Lou 2011
Wang 2011
D1: Random sequence generation
D2: Allocation concealment
D3: Blinding of participants and personnel
D4: Blinding of outcome assessment
D5: Incomplete outcome data
D6: Selective reporting
D7: Other bias
Judgement
Low
Unclear
High

(Fig. S1)

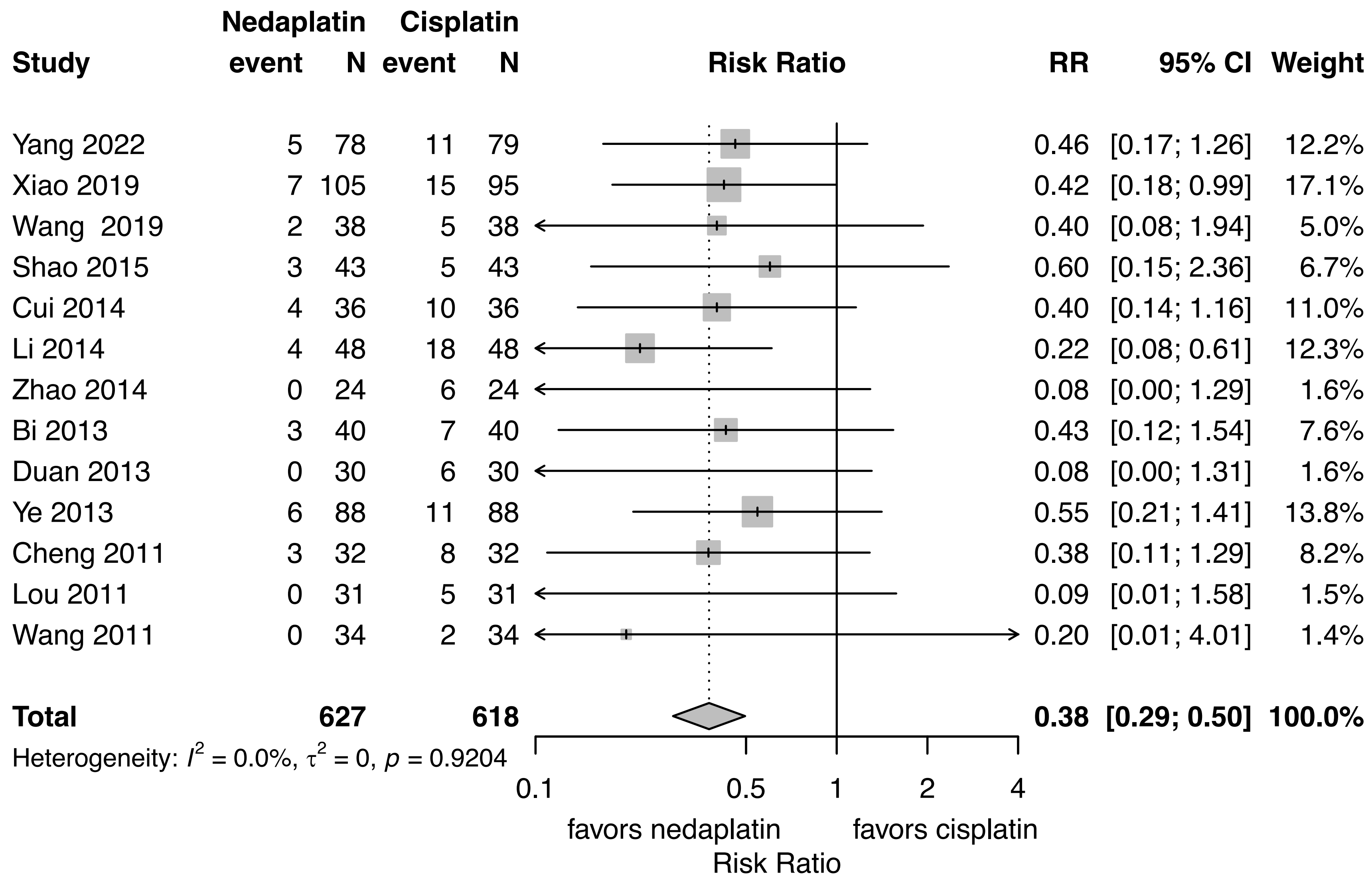
Nedaplatin
Cisplatin
Study event N event N
Risk Ratio
RR 95% CI Weight
Yang 2022 5 78 11 79 0.46 [0.17; 1.26] 12.2%
Xiao 2019 7 105 15 95 0.42 [0.18; 0.99] 17.1%
Wang 2019 2 38 5 38 0.40 [0.08; 1.94] 5.0%
Shao 2015 3 43 5 43 0.60 [0.15; 2.36] 6.7%
Cui 2014 4 36 10 36 0.40 [0.14; 1.16] 11.0%
Li 2014 4 48 18 48 0.22 [0.08; 0.61] 12.3%
Zhao 2014 0 24 6 24 0.08 [0.00; 1.29] 1.6%
Bi 2013 3 40 7 40 0.43 [0.12; 1.54] 7.6%
Duan 2013 0 30 6 30 0.08 [0.00; 1.31] 1.6%
Ye 2013 6 88 11 88 0.55 [0.21; 1.41] 13.8%
Cheng 2011 3 32 8 32 0.38 [0.11; 1.29] 8.2%
Lou 2011 0 31 5 31 0.09 [0.01; 1.58] 1.5%
Wang 2011 0 34 2 34 0.20 [0.01; 4.01] 1.4%
Total 627 618 0.38 [0.29; 0.50] 100.0%
Heterogeneity: I^2 = 0.0%, τ^2 = 0, p = 0.9204
0.1
0.5
1
2
4
favors nedaplatin
favors cisplatin
Risk Ratio

(Fig. S2)

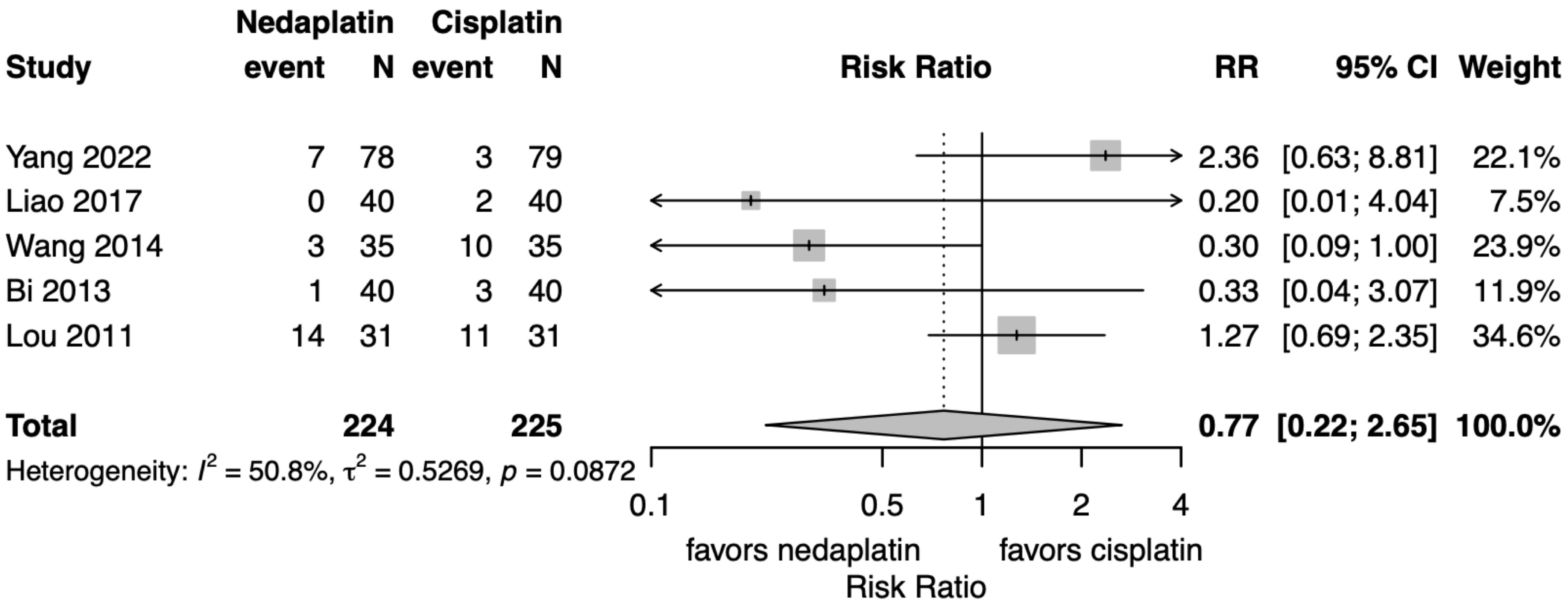
Nedaplatin
Cisplatin
Study
event
N
event
N
Risk Ratio
RR
95% CI
Weight
Yang 2022 7 78 3 79 2.36 [0.63; 8.81] 22.1%
Liao 2017 0 40 2 40 0.20 [0.01; 4.04] 7.5%
Wang 2014 3 35 10 35 0.30 [0.09; 1.00] 23.9%
Bi 2013 1 40 3 40 0.33 [0.04; 3.07] 11.9%
Lou 2011 14 31 11 31 1.27 [0.69; 2.35] 34.6%
Total 224 225 0.77 [0.22; 2.65] 100.0%
Heterogeneity: $I^2 = 50.8\%$, $\tau^2 = 0.5269$, $p = 0.0872$
0.1
0.5
1
2
4
favors nedaplatin
favors cisplatin
Risk Ratio

(Fig. S3)

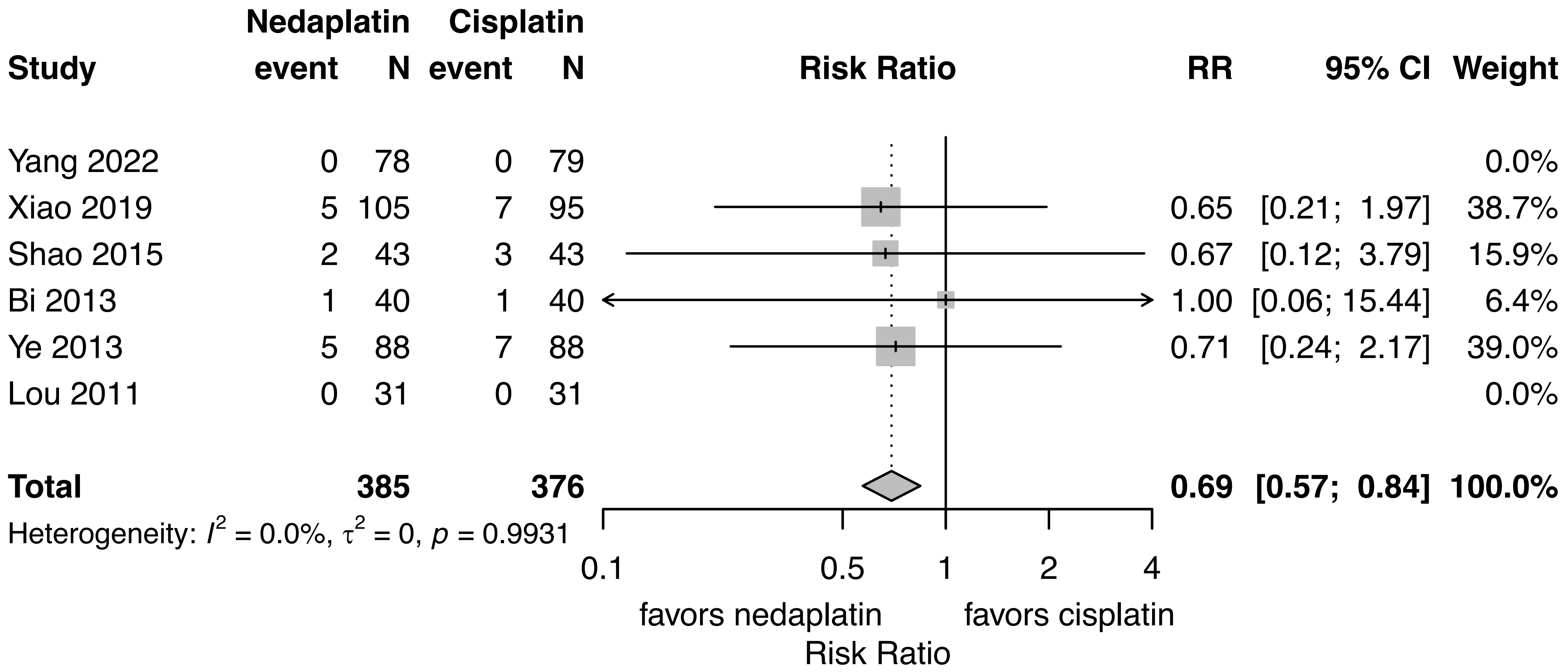
Nedaplatin
Cisplatin
Study
event
N
event
N
Risk Ratio
RR
95% CI
Weight
Yang 2022 0 78 0 79 0.0%
Xiao 2019 5 105 7 95 0.65 [0.21; 1.97] 38.7%
Shao 2015 2 43 3 43 0.67 [0.12; 3.79] 15.9%
Bi 2013 1 40 1 40 1.00 [0.06; 15.44] 6.4%
Ye 2013 5 88 7 88 0.71 [0.24; 2.17] 39.0%
Lou 2011 0 31 0 31 0.0%
Total 385 376 0.69 [0.57; 0.84] 100.0%
Heterogeneity: I^2 = 0.0%, τ^2 = 0, p = 0.9931
0.1
0.5
1
2
4
favors nedaplatin
favors cisplatin
Risk Ratio

(Fig. S4)

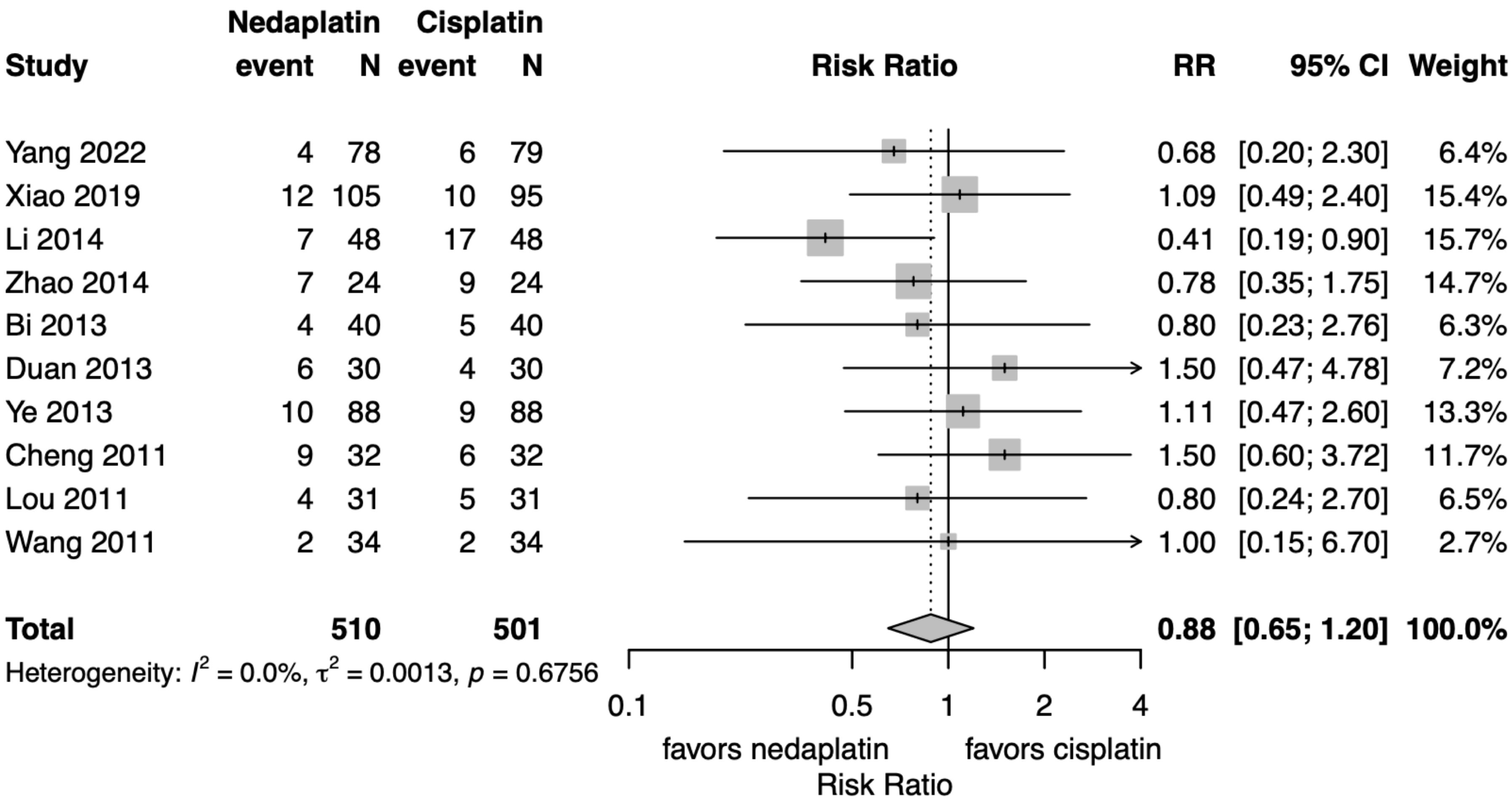
Nedaplatin Cisplatin
Study event N event N Risk Ratio RR 95% CI Weight
Yang 2022 4 78 6 79 0.68 [0.20; 2.30] 6.4%
Xiao 2019 12 105 10 95 1.09 [0.49; 2.40] 15.4%
Li 2014 7 48 17 48 0.41 [0.19; 0.90] 15.7%
Zhao 2014 7 24 9 24 0.78 [0.35; 1.75] 14.7%
Bi 2013 4 40 5 40 0.80 [0.23; 2.76] 6.3%
Duan 2013 6 30 4 30 1.50 [0.47; 4.78] 7.2%
Ye 2013 10 88 9 88 1.11 [0.47; 2.60] 13.3%
Cheng 2011 9 32 6 32 1.50 [0.60; 3.72] 11.7%
Lou 2011 4 31 5 31 0.80 [0.24; 2.70] 6.5%
Wang 2011 2 34 2 34 1.00 [0.15; 6.70] 2.7%
Total 510 501 0.88 [0.65; 1.20] 100.0%
Heterogeneity: I^2 = 0.0%, τ^2 = 0.0013, p = 0.6756
0.1 0.5 1 2 4
favors nedaplatin favors cisplatin
Risk Ratio

(Fig. S5)

Study	Nedaplatin event	Nedaplatin N	Cisplatin event	Cisplatin N	RR	95% CI	Weight
Yang 2022	0	78	0	79			0.0%
Xiao 2019	10	105	3	95	3.02	[0.86; 10.63]	14.6%
Shao 2015	3	43	5	43	0.60	[0.15; 2.36]	13.5%
Li 2014	8	48	18	48	0.44	[0.21; 0.92]	20.8%
Zhao 2014	0	24	0	24			0.0%
Bi 2013	2	40	0	40	5.00	[0.25; 100.93]	4.7%
Duan 2013	12	30	5	30	2.40	[0.96; 5.98]	18.6%
Ye 2013	7	88	10	88	0.70	[0.28; 1.76]	18.5%
Cheng 2011	2	32	0	32	5.00	[0.25; 100.15]	4.7%
Wang 2011	2	34	0	34	5.00	[0.25; 100.37]	4.7%
**Total**		**522**		**513**	**1.28**	**[0.57; 2.88]**	**100.0%**

Heterogeneity: $I^2 = 55.3\%$, $\tau^2 = 0.5053$, $p = 0.0285$

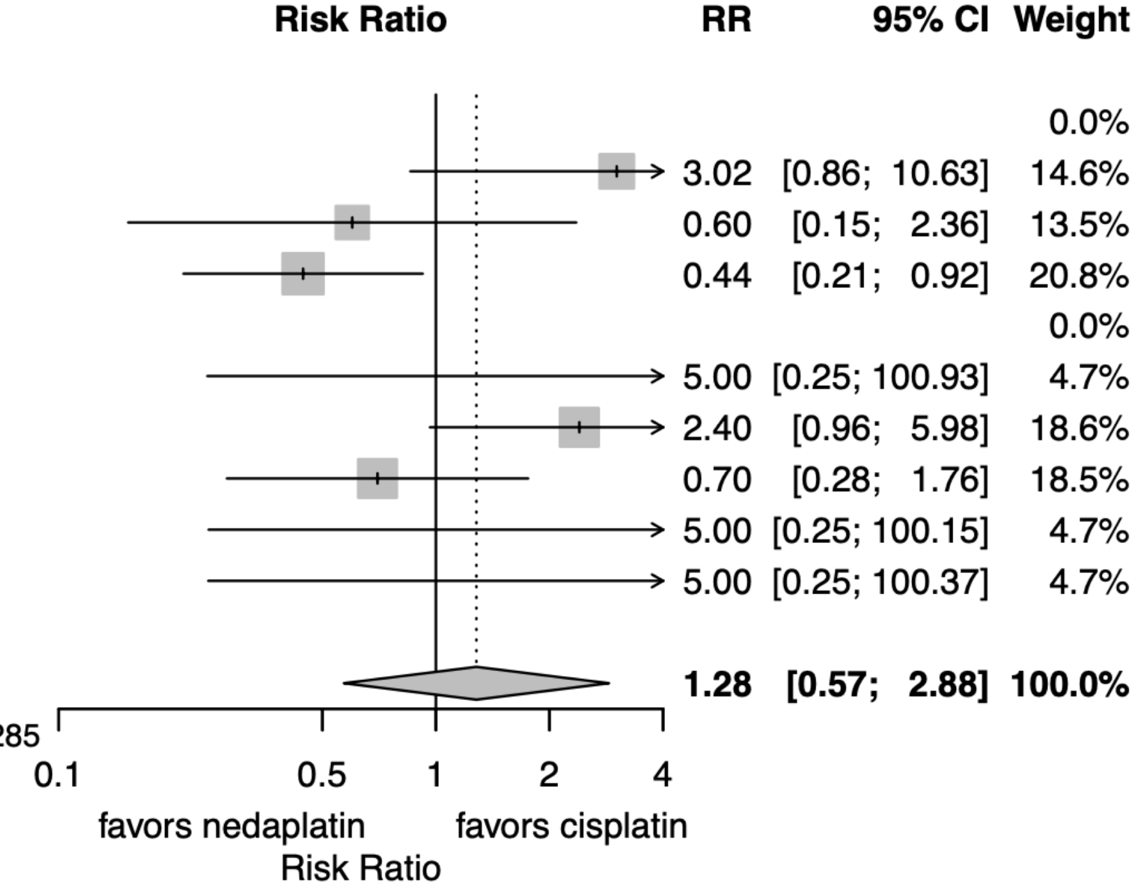

(Fig. S6)
