# Supplementary material for: Comparison of nedaplatin and cisplatin in concurrent chemoradiotherapy for cervical cancer: a systematic review and meta-analysis
Source: Int J Clin Oncol. 2026 Jan 29;31(3):537–47. doi: 10.1007/s10147-026-02968-6 (PMC12932270; doi:10.1007/s10147-026-02968-6)
Supplement: Supplementary file 2 — Supplementary file2 (DOCX 18 KB) [file 10147_2026_2968_MOESM2_ESM.docx]

(Table S1.)

|  | **ovid MEDLINE** | **Results from 27 Mar 2025** |
| --- | --- | --- |
| 1 | exp Uterine Cervical Neoplasms/ | 90,172 |
| 2 | (cervi* adj5 (cancer* or neoplas* or carcinom* or malignan* or tumor* or tumour*)).mp. | 136,430 |
| 3 | 1 or 2 | 136,430 |
| 4 | exp Antineoplastic Agents/ | 1,312,658 |
| 5 | Chemoradiotherapy/ | 18,044 |
| 6 | chemotherap*.mp. | 639,751 |
| 7 | radiotherapy/ | 44,792 |
| 8 | radiotherap*.mp. | 402,637 |
| 9 | radiation/ | 9,579 |
| 10 | (chemoradi* or radiochemo).mp. | 48,830 |
| 11 | 4 or 5 or 6 or 7 or 8 or 9 or 10 | 1,966,389 |
| 12 | cisplatin.mp. | 93,432 |
| 13 | cis-Diamminedichloroplatinum.mp. | 2,308 |
| 14 | Neoplatin.mp. | 4 |
| 15 | cddp.mp. | 8,740 |
| 16 | cis-ddp.mp. | 631 |
| 17 | 12 or 13 or 14 or 15 or 16 | 94,758 |
| 18 | nedaplatin.mp. | 912 |
| 19 | NSC375101.mp. | 0 |
| 20 | CDGP.mp. | 196 |
| 21 | Aqupla.mp. | 2 |
| 22 | 254-S.mp. | 154 |
| 23 | diammineplatinum II.mp. | 94 |
| 24 | 18 or 19 or 20 or 21 or 22 or 23 | 1,225 |
| 25 | randomized controlled trial.mp. | 689,151 |
| 26 | controlled clinical trial.mp. | 116,446 |
| 27 | randomized.ab. | 684,256 |
| 28 | randomly.ab. | 455,759 |
| 29 | trial.ti. | 331,465 |
| 30 | 25 or 26 or 27 or 28 or 29 | 1,466,381 |
| 31 | 3 and 11 and 17 and 24 and 30 | 9 |
|  |  |  |
|  | **CENTRAL** |  |
| 1 | [mh “Cervical Neoplasms”] | 3,445 |
| 2 | (cervi* near/5 (cancer* or neoplas* or carcinom* or malignan* or tumor* or tumour*)) | 8,552 |
| 3 | #1 or #2 | 8,552 |
| 4 | cisplatin | 17,157 |
| 5 | cis-Diamminedichloroplatinum | 52 |
| 6 | neoplatin | 11 |
| 7 | cddp | 1,131 |
| 8 | cis-ddp | 22 |
| 9 | #4 or #5 or #6 or #7 or #8 | 17,509 |
| 10 | nedaplatin | 225 |
| 11 | NSC 375101 | 0 |
| 12 | CDGP | 24 |
| 13 | Aqupla | 1 |
| 14 | 254 S | 4,935 |
| 15 | diammineplatinum II | 7 |
| 16 | #10 or #11 or #12 or #13 or #14 or #15 | 5,188 |
| 17 | [mh “Radiotherapy”] | 9,836 |
| 18 | radiotherap* | 44,358 |
| 19 | radiation | 39,743 |
| 20 | (chemoradi* or radiochemo*) | 11,714 |
| 21 | #17 or #18 or #19 or #20 | 65,764 |
| 22 | #3 and #9 and #16 and #21 | 14 |
|  |  |  |
|  | **clinicaltrials.gov** | 416 |
| 1 | Condition or disease: uterine cervical cancer |  |
| 2 | Intervention/treatment: chemoradiatherapy |  |
| 3 | Study type: Interventional Studies | 179 |
|  |  |  |
|  | **WHO ICTRP** |  |
|  | cervical cancer AND chemoradiotherapy | 128 |
|  |  |  |
|  | **Embase** |  |
| 1 | emb(Cervical Neoplasms) | 1763 |
| 2 | cervi* NEAR/5 cancer* or neoplas* or carcinom* or malignan* or tumor* or tumour* | 6,139,322 |
| 3 | 1 or 2 | 6,139,322 |
| 4 | antineoplastic agents | 1,400 |
| 5 | chemoradiotherapy | 103,580 |
| 6 | chemotherap* | 1,296,676 |
| 7 | radiotherapy | 846,112 |
| 8 | radiotherap* | 855,071 |
| 9 | radiation | 1,455,554 |
| 10 | chemoradi* or radiochemo* | 114,460 |
| 11 | 4 or 5 or 6 or 7 or 8 or 9 or 10 | 2,835,485 |
| 12 | cisplatin | 248,066 |
| 13 | cis-Diamminedichloroplatinum | 2,473 |
| 14 | neoplatin | 41 |
| 15 | cddp | 11,535 |
| 16 | cis-ddp | 665 |
| 17 | 12 or 13 or 14 or 15 or 16 | 249,740 |
| 18 | nedaplatin | 3,243 |
| 19 | nsc 375101 | 0 |
| 20 | cdgp | 305 |
| 21 | aqupla | 18 |
| 22 | 254-S | 214 |
| 23 | diammineplatinum II | 124 |
| 24 | 18 or 19 or 20 or 21 or 22 or 23 | 3,643 |
| 25 | randomized controlled trial | 1,286,258 |
| 26 | controlled clinical trial | 1,719,968 |
| 27 | ab(randomized) | 995,033 |
| 28 | ab(randomly) | 608,181 |
| 29 | ti(trial) | 461,411 |
| 30 | 25 or 26 or 27 or 28 or 29 | 2,613,975 |
| 31 | 3 and 9 and 16 and 24 and 30 | 290 |
|  |  |  |
|  | **CNKI** |  |
| 1 | Subject：cervical cancer |  |
| 2 | Title, keyword and abstract = nedaplatin |  |
| 3 | Title, keyword and abstract = radiation | 82 |
|  |  |  |
|  | **Ichushi web** |  |
| 1 | “Cervical cancer” OR “Cervical neoplasms” | 45,670 |
| 2 | “Nedaplatin” OR “ネダプラチン” | 2,654 |
| 3 | “Radiotherapy” OR “放射線” | 159,395 |
|  | #1 and #2 and #3 | 62 |
